# The association between neonatal death and facility birth in regions of India

**DOI:** 10.4054/demres.2019.40.16

**Published:** 2019-02-26

**Authors:** Diane Coffey

**Affiliations:** 1 Population Research Center and Department of Sociology, University of Texas, Austin, USA; r.i.c.e – Research Institute for Compassionate Economics, Amston, USA; Indian Statistical Institute, Delhi Centre, New Delhi, India.

## Abstract

**BACKGROUND:**

Reducing neonatal mortality in India is critical to achieving the 2030 Sustainable Development Goal of a global neonatal mortality rate (NNM) of no more than 12 per 1,000. Policy efforts to reduce India’s NNM, including a large-scale conditional cash transfer program, have focused on promoting birth in health facilities, rather than at home. Between 2005 and 2015, the percentage of facility births doubled, from 40% to 80%.

**OBJECTIVE:**

We assess evidence for the hypothesis that facility births reduce NNM by using new data from the National Family Health Survey, 2015–2016.

**METHODS:**

We analyze the association between neonatal death and facility birth at the region level, using ordinary least squares (OLS) linear probability models with fixed effects for the primary sampling unit, as well as child, mother, and household-level controls.

**RESULTS:**

For babies born outside of Uttar Pradesh and Bihar, facility birth is robustly associated with neonatal survival. The controlled association between facility birth and neonatal survival is 7 per 1,000 in the east region (West Bengal, Assam, Jharkhand, Odisha) and 13 per 1,000 in the central region (Madhya Pradesh and Chhattisgarh). In Uttar Pradesh and Bihar, however, being born in a health facility appears to confer no neonatal survival advantage.

**CONTRIBUTION:**

Documenting the lack of an association between facility birth and neonatal death in Uttar Pradesh and Bihar is important because these states collectively contribute 43% of India’s NNM. These findings suggest the need for future research to investigate whether and how the quality of maternal and newborn care in health facilities differs across regions.

## 1. Introduction

The United Nations’ Sustainable Development Goal 3 aims to reduce global neonatal mortality (NNM) to 12 deaths per 1,000 live births by 2030. Reducing NNM in India is critical to achieving this goal because it is home to 27% of neonatal deaths but only 19% of births. India contributes more neonatal deaths than any other country. Its national NNM of 30 per 1,000 in 2015 (IIPS and ICF 2017) masks wide variation across places. Among states with more than 25 million people, Uttar Pradesh had the highest NNM at 45 per 1,000, and Kerala had the lowest at 4 per 1,000. India’s state-level variation in NNM is similar to the country-level variation in NNM that exists on a global scale. According to the 2015 World Development Indicators, the Central African Republic had the second highest NNM in the world at 43 per 1,000, and the United States had an NNM of 4 per 1,000 (World Bank Group 2015).

India’s high NNM is particularly surprising in light of a recent, dramatic increase in the fraction of births that occur in health facilities, rather than at home. In 2005, about 40% of births took place in health facilities; by 2015, this figure was 80%. The increase in facility births was in part due to a large-scale, conditional cash transfer program called *Janani Surkasha Yojana* (JSY). JSY was launched in 2005 as part of the central government’s new National Rural Health Mission (NRHM). JSY, which means Safe Motherhood Scheme, pays local health workers to accompany women to deliver in health facilities. Women who deliver in health facilities also receive a cash payment.

A key assumption of the JSY program, and of much of the Indian government’s maternal and child health strategy, is that shifting births from homes to health facilities will reduce NNM (Rao 2017). However, causal analysis to test the validity of this assumption is difficult; it is not possible to randomly assign place of birth.

Can descriptive analysis help deepen our understanding of the relationship between NNM and facility birth? The uncontrolled association, at the individual level, between neonatal death and facility birth is likely to be confounded by a number of factors. For instance, in India’s National Family Health Survey (NFHS)-3, collected before JSY was implemented in 2005, there was little difference in neonatal survival between babies born at home and those born in health facilities. The lack of association could have had many possible explanations, among them that health facilities were ineffective at promoting neonatal health, or that the mothers who delivered in health facilities were a mix of privileged women (whose neonates may have survived regardless of where they were born) and women with labor complications (who may often have arrived too late to be helped by the health facility). Considering the large variation in quality of public services across regions and states of India (Drèze and Sen 2013), it is also possible that the true effect of` hospital birth on neonatal survival was heterogenous.

In this descriptive paper, I use the latest-available data from the National Family Health Survey, 2015–2016 (NFHS-4) to advance understanding of the association between neonatal survival and facility birth. The weighted, uncontrolled, all-India association between neonatal survival and facility birth was 12 per 1,000. That is, NNM was 12 per 1,000 lower among children born in health facilities than among children born at home. The analysis that follows unpacks this association at the region level, showing that it is robust to controls for possible omitted factors in some regions – particularly in east and central India – and not statistically significantly different from zero in others, including in Uttar Pradesh and Bihar, two states that together contribute about 43% of India’s NNM.

## 2. Data and methods

### 2.1 Data

NFHS-4, conducted between January 2015 and December 2016 is India’s most recent Demographic and Health Survey (DHS). The prior DHS round, the NFHS-3, was conducted in 2005–2006. Both are nationally representative, multistage, clustered sample surveys. This paper’s analyses focus on the 2015–2016 survey; summary statistics from both surveys are used to identify recent trends.

States with populations of over 25 million at the time of the 2011 Census are included in the analyses. These states are home to approximately 95% of the population of India. States are grouped into regions based on the NFHS-4’s regional classifications (IIPS and ICF 2017). Table 1 shows the division of states into regions. Uttar Pradesh and Bihar are treated as their own region, the focus region, because these geographically contiguous states have high NNM and contribute a disproportionate number of neonatal deaths.

#### 2.1.1 Dependent variable

The dependent variable of interest is neonatal death. It is coded as either “0” if the child survived the first month of life, or “1,000” if the child died in the first month of life. Coding death as “1,000” rather than “1” for the regression analysis does not change the results; it simply changes the scale of regression coefficients so that they are easy to interpret as effects on “per 1,000” rates, which is how NNM is normally published. Children born in the five years before the survey are included in the sample, except for children who were born less than a month before the survey, as their neonatal survival status is unknown.

Table 1 provides state and region-level estimates of NNM that are computed using the method that the DHS uses to compute published summary statistics (Rutstein and Rojas 2006). Table 1 also provides information about the percentage of India’s births and neonatal deaths that occur in each region and state. It shows that the states of Uttar Pradesh and Bihar contribute disproportionately to neonatal death relative to births.

#### 2.1.2 Independent variable

The independent variable of interest is an indicator that is equal to “1” if the child’s birth took place in a health facility (public or private) and “0” if the birth took place at home. The NFHS-4 collected information on place of birth for live births that occurred in the five years before the survey.^2^ Table 1 summarizes state and region-level variation in the fraction of births that took place in health facilities. It also includes statistics from the NFHS-3 for comparison.

**Figure 1:**
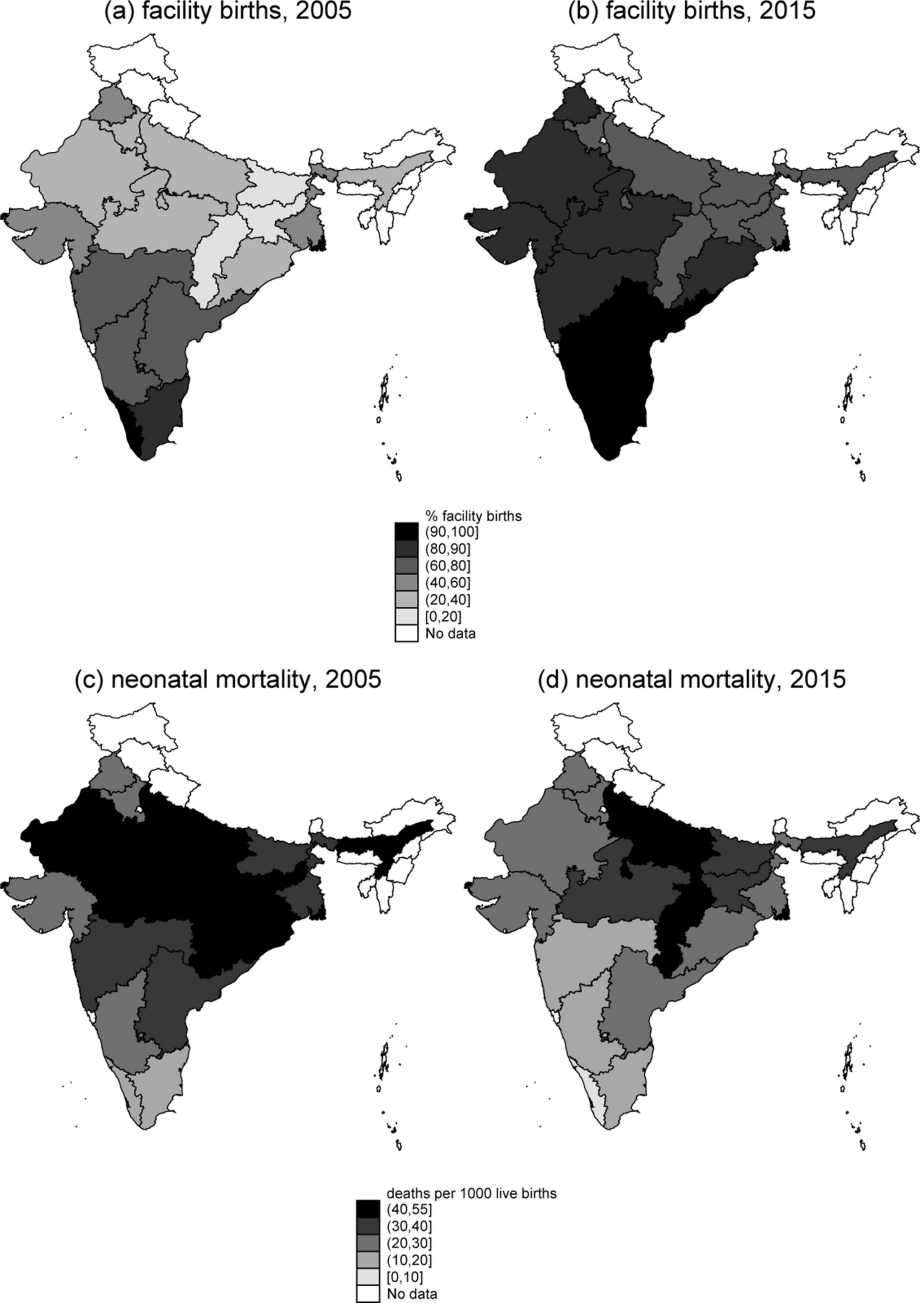
Map of changes in facility birth and NNM in Indian states with greater than 25 million population *Note*: Panels a and b show the percentage of births in the five years before the survey that took place in a health facility, for 2005 and 2015 respectively. Panels c and d show the neonatal mortality rate, in deaths per 1,000 live births, for 2005 and 2015 respectively.

Figure 1 shows maps that depict how the fraction of births that take place in a health facility changed between 2005 (panel a) and 2015 (panel b) for the states with more than 25 million population in 2011. Panels c and d of Figure 1 depict changes in NNM for the same set of states.

#### 2.1.3 Control variables

Table 1 shows large variation in NNM across places in India, which suggests that NNM may be influenced by a number of variables other than facility birth, such as the disease environment, underlying maternal health, levels of gender empowerment, cultural practices, and access to prenatal services. Our primary strategy for controlling for these and other possible confounding variables is to use fixed effects for a child’s primary sampling unit (PSU). PSUs are villages in rural areas and census enumeration blocks in urban areas (IIPS and ICF 2017). Children in the same PSUs share many of the characteristics described above. The coefficient on facility birth that is produced by using a PSU-fixed effects regression is computed by averaging differences in neonatal survival between children who were born in health facilities and at home within the same PSU. PSUs in which there is no variation in place of birth do not contribute to the estimate.^3^

A PSU-fixed effects regression also permits child, mother, and household-level demographic and socioeconomic controls. Below, we describe the wide array of additional controls included in the model.

### 2.2 Methods

**Figure 2:**
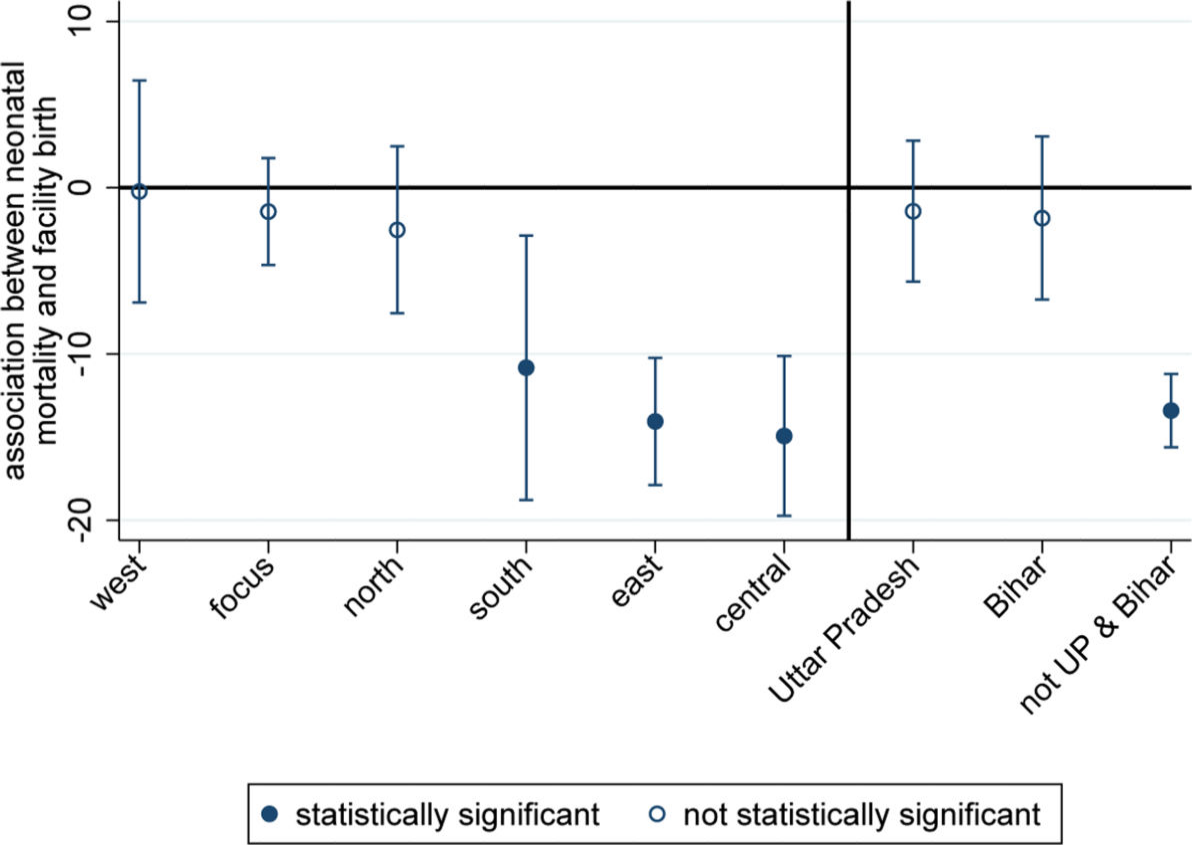
Associations between NNM and facility birth, no controls, 2015–2016 *Note*: The figure plots OLS regression coefficients and confidence intervals (computed with standard errors clustered by PSU) from regressions of NNM on facility birth (see Equation 1) for regions and states of India. Gujarat and Maharastra compose ‘west’; Uttar Pradesh and Bihar compose ‘focus’; Haryana, Punjab, and Rajasthan compose ‘north’; Andhra Pradesh, Telegana, Karnataka, Kerala, and Tamil Nadu compose ‘south’; Orissa, West Bengal, Jharkhand, and Assam compose ‘east’; and Madhya Pradesh and Chhattisgarh compose ‘central.’ ‘Not UP & Bihar’ indicates a result that pools states in all other regions except for Uttar Pradesh and Bihar.

**Figure 3:**
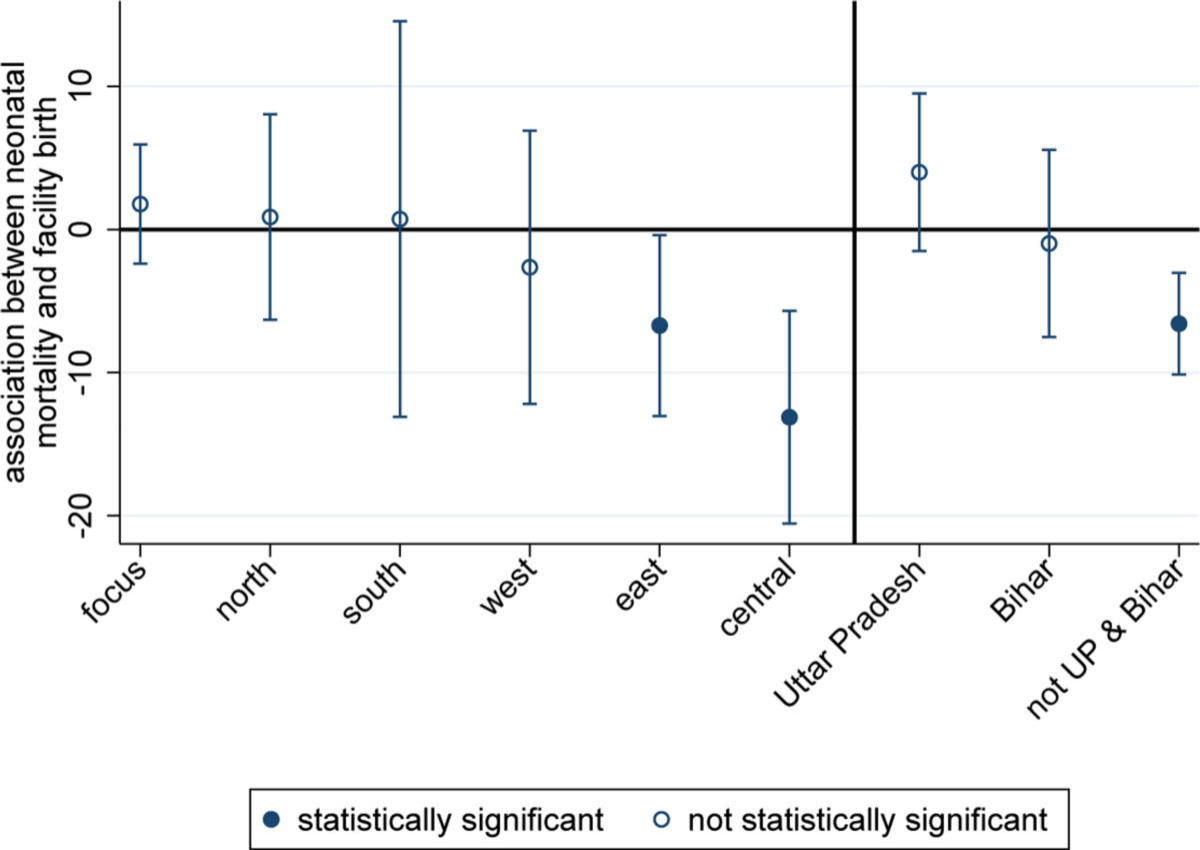
Associations between NNM and facility birth, with controls, 2015–2016 *Note*: The figure plots OLS regression coefficients and confidence intervals (computed with standard errors clustered by PSU) from regressions of NNM on facility birth and controls (see Equation 2 and Section 2.2 for a description of the controls) for regions and states of India. The note for Figure 2 lists the states that correspond to each region.

Figures 2 and 3 present the results of uncontrolled and controlled regressions respectively. Figure 2 plots coefficients and standard errors from region or state-level regressions of the following form:

(1) $$neonatal\;death_{i}=\beta_{0}+\beta_{1}facility\;birth_{i}+\epsilon_{i}$$

where i indexes live births and ${\hat{\beta }}_{1}$ is the estimated coefficient of interest.

Figure 3 plots coefficients and standard errors from region or state-level regressions of the following form:

(2) $$\begin{array}{l} neonatal\;death_{ip}=\beta_{0}+\beta_{1}facility\;birth_{ip}+\beta_{2}male_{ip}+\sum_{b=1}^{b=4^{+}}\beta_{3}^{b}birthorder_{ip}\\ +\sum_{s=1}^{s=6^{+}}\beta_{4}^{s}sibsize_{ip}+\beta_{5}mother^{\prime }s\;years\;ofeducation_{ip}\\ +\beta_{6}mother^{\prime }s\;age\;at\;birth_{ip}\text{+}\beta_{\text{7}}mother^{\prime }s\;age\;at\;birth_{ip}^{\text{2}}\\ +\alpha_{p}+M_{ip}\theta +SES_{ip}\gamma +\epsilon_{ip}, \end{array}$$

where $\alpha_{p}$ are fixed effects for child i’s primary sampling unit, p; $M_{ip}$ is a vector of fixed effects for the century month code (CMC) of the child’s birth; and $SES_{ip}$ is a large vector of indicators for household socioeconomic status.^4^ And $male_{ip}$ controls for the sex of the child;^5^ birth order is entered as indicator variables, ranging from 1 to 4^+^; and sibsize is a set of indicators for the number of children ever born to the child’s mother at the time of the survey, ranging from 1 to 6^+^. The model also includes controls for the mother’s education in years, and mother’s age and age-squared (both in years) at the time of child i’s birth. Any survival advantage of being born in a health facility that remains after controlling for these indicators reflects a difference that persists even after very detailed demographic, geographic, and SES information has been accounted for.

## 3. Results

### 3.1 Association between neonatal death and facility birth, by region

Figure 2 plots coefficients and standard errors from Equation 1. It documents associations between neonatal death and facility birth, without controls, in different regions of India. In the south, east, and central regions, there is a negative association between neonatal death and facility birth. In the focus, north, and west regions, babies born in health facilities are no more likely to survive than those born at home.

### 3.2 Within-village comparisons, by region

Section 2 suggests several reasons why the coefficient on facility birth estimated by Equation 1 may be downwardly biased. Figure 3 plots coefficients on facility birth that are estimated using Equation 2, which controls for PSU fixed effects, as well as a host of child, mother, and household-level demographic and socioeconomic characteristics. For the south region, the introduction of controls attenuates the coefficient to the point that it is no longer statistically significant, suggesting that women who deliver at home in the south may be different, on average, from the majority who deliver in health facilities.^6^ In east and central India, in contrast, the association between neonatal death and facility birth is both negative and statistically significant, even after the introduction of this large set of controls.

Perhaps the most striking result presented in Figure 3 is given on the right side of the figure. In a pooled regression using data from all of the regions except the focus region – Uttar Pradesh and Bihar – the association between neonatal death and facility birth is negative and statistically significant. For Bihar, the coefficient is not statistically significant, but it is slightly negative. In contrast, the point estimate for Uttar Pradesh is positive, and the confidence interval on the estimate does not overlap with that for the pooled estimate for the other regions.

For each of the three places on the right side of Figure 3, Table 2 shows coefficient estimates for facility birth as well as many of the control variables included in the model described by Equation 2. The coefficient estimates underscore the importance of the controls – especially birth order and sibsize – in predicting neonatal mortality in all three places.^7^ Further, children whose mothers are at the extremes of the age distribution, and male children, are more likely to die neonatal deaths. However, it is noteworthy that, outside of Uttar Pradesh and Bihar, even after accounting for this wide array of child, mother, and household-level demographic and socioeconomic controls, as well as PSU fixed effects, children born in a health facility face an NNM about 7 per 1,000 lower than children born at home.

## 4. Discussion

Considering that promoting facility birth has been the cornerstone of maternal and newborn health policy in India for much of the last decade, the finding that facility birth is uncorrelated with neonatal death in Uttar Pradesh and Bihar – which together contribute 43% of India’s neonatal mortality – is concerning. It is especially concerning for the state of Uttar Pradesh, which has a 2015 neonatal mortality rate higher than every country in the world except Pakistan, and which contributes 27% of India’s neonatal deaths.

The lack of an association between neonatal death and facility birth coheres with prior qualitative research which suggests that the quality of maternal and newborn care in health facilities in this region is extremely poor (Jeffery and Jeffery 2010; Coffey 2014). It also coheres with the results of Semrau et al. (2017), a randomized controlled trial of the “Better Birth” coaching program which aimed to improve the quality of maternal and newborn care in health facilities in 24 districts of Uttar Pradesh. The program, which was successful in other contexts (Kabongo et al. 2017), was intensive. It consisted of 43 day-long coaching visits to each facility over a period of eight months. Nevertheless, twelve months after the program was implemented, researchers found only modest differences in the quality of maternal and newborn care provided in intervention vs. control facilities. They found no differences in perinatal or maternal mortality, nor in major health complications following delivery. Thus, both qualitative and quantitative research provide evidence that the government’s rather singular focus on promoting facility birth may be misguided in this high-mortality region.

The robust, negative association between neonatal death and facility birth in the east and central regions is, however, encouraging, and suggests that the JSY program may have had heterogenous effects. These regions saw much larger improvements in NNM in the last decade than were observed for Uttar Pradesh and Bihar. Robust associations between neonatal death and facility birth are consistent with evidence from the Million Deaths Study, which studied changes in causes of death between 2000 and 2015 in partnership with India’s Sample Registration System (Fadel et al. 2017). The study found that declines in NNM came primarily from declines in birth trauma and birth asphyxia, causes of neonatal death that could plausibly be influenced by care at birth in a health facility. We note that it is puzzling that Fadel et al. (2017) find significant declines in neonatal mortality from birth asphyxia and birth trauma in Bihar and Uttar Pradesh, as well as in the central and east regions. Future research might usefully compare how delivery and postpartum care practices differ between the central and east regions and the states of Uttar Pradesh and Bihar.

## Figures and Tables

**Table 1:** Place of birth and NNM in Indian states, 2005–2006 and 2015–2016

	Fraction		Fraction		NNM^b^		Fraction of India’s total^c^			
	Home birth^a^		Facility birth^a^				Births		Neonatal deaths	
Region and state	05–06	15–16	05–06	15–16	05–06	15–16	05–06	15–16	05–06	15–16

**Focus states**	0.80	0.34	0.20	0.66	45	42	0.32	0.30	0.37	0.43
Uttar Pradesh	0.79	0.32	0.21	0.68	48	45	0.21	0.18	0.25	0.27
Bihar	0.80	0.36	0.20	0.64	40	37	0.11	0.13	0.11	0.16
**Central**	0.77	0.20	0.23	0.80	46	38	0.09	0.10	0.11	0.12
Madhya Pradesh	0.74	0.19	0.26	0.81	45	37	0.07	0.07	0.08	0.08
Chhattisgarh	0.86	0.30	0.14	0.70	51	42	0.02	0.02	0.03	0.03
**East**	0.67	0.26	0.33	0.74	43	27	0.17	0.17	0.18	0.14
West Bengal	0.58	0.24	0.42	0.76	38	22	0.07	0.07	0.07	0.05
Jharkhand	0.82	0.38	0.18	0.62	49	33	0.03	0.03	0.04	0.03
Assam	0.77	0.29	0.23	0.71	46	33	0.03	0.02	0.03	0.03
Odisha	0.64	0.14	0.36	0.86	45	28	0.03	0.03	0.04	0.03
**West**	0.40	0.10	0.60	0.90	32	20	0.13	0.12	0.10	0.08
Maharashtra	0.35	0.10	0.65	0.90	32	16	0.08	0.09	0.06	0.05
Gujarat	0.47	0.11	0.53	0.89	34	27	0.05	0.04	0.04	0.04
**South**	0.26	0.05	0.74	0.95	29	17	0.16	0.18	0.12	0.10
Andhra Pradesh and Telegana	0.35	0.08	0.65	0.92	40	22	0.06	0.06	0.06	0.05
Tamil Nadu	0.12	0.01	0.88	0.99	19	14	0.04	0.06	0.02	0.03
Karnataka	0.35	0.06	0.65	0.94	29	19	0.05	0.04	0.03	0.03
Kerala	0.01	0.00	0.99	1.00	12	4	0.02	0.02	0.01	0.00
**North**	0.65	0.16	0.35	0.84	37	27	0.10	0.10	0.10	0.10
Harayana	0.64	0.20	0.36	0.80	24	22	0.02	0.02	0.01	0.02
Punjab	0.49	0.10	0.51	0.90	28	21	0.02	0.02	0.01	0.01
Rajasthan	0.70	0.16	0.30	0.84	44	30	0.06	0.06	0.07	0.06

*Note*: Summary statistics are shown for Indian states that had populations larger than 25 million in the 2011 Census of India. In each survey year, these states accounted for 97% of NNM in India. Estimates use survey weights provided by the NFHS.

a A ‘home birth’ is a birth that occurred at home; a ‘facility birth’ is a birth that occurred in a health facility. The NFHS collected data on place of birth for each mother’s last three births in the five years before the survey.

b NNM is neonatal mortality, the number of deaths per 1,000 live births that took place in the first month of life. NNM is computed by using the same method that is used by the DHS (see Rutstein and Rojas 2006) and uses births in the five years before the survey.

c Figures for the percentage of total births and neonatal deaths do not add to one because they use all of India in the denominator, not states with populations greater than 25 million. These figures are computed using births in the five years before the survey.

**Table 2:** Predictors of neonatal mortality, 2015–2016

Dependent variable:	Neonatal death = 1,000; neonatal survival = 0		
	Uttar Pradesh	Bihar	Other regions^a^

Facility	3.997	−0.979	−6.580***
	(2.803)	(3.335)	(1.814)
Male	11.11***	13.99***	11.11***
	(2.291)	(2.568)	(0.997)
Birth order 2	−55.61***	−48.60***	−44.69***
	(4.426)	(5.443)	(2.011)
Birth order 3	−102.2***	−80.23***	−98.67***
	(7.037)	(8.666)	(3.794)
Birth order 4	−151.4***	−119.7***	−149.6***
	(10.38)	(12.03)	(6.135)
Sibsize of 2	50.50***	42.61***	40.91***
	(4.743)	(6.026)	(2.125)
Sibsize of 3	104.6***	75.20***	96.99***
	(7.267)	(9.125)	(3.806)
Sibsize of 4	164.6***	123.7***	151.7***
	(10.17)	(12.39)	(6.176)
Sibsize of 5	187.1***	135.4***	163.5***
	(12.12)	(14.17)	(7.155)
Sibsize of 6	198.3***	151.6***	180.9***
	(12.34)	(14.39)	(7.751)
Mother’s education (in years)	−2.089	−4.991*	−0.339
	(1.583)	(1.965)	(0.496)
Mother’s age at birth	−7.934***	−8.150**	−3.678***
	(2.188)	(2.688)	(0.985)
Mother’s age at birth^2^	0.132***	0.134**	0.0680***
	(0.0386)	(0.0492)	(0.0183)
PSU fixed effects	✓	✓	✓
CMC fixed effects	✓	✓	✓
SES indicators	✓	✓	✓
*n*	40,256	24,721	139,267

*Note*: The table reports OLS regression coefficients and standard errors (clustered by PSU) from regressions of NNM on facility birth and controls (see Equation 2 and Section 2.2 for a description of the controls).

* p < 0.05

** p < 0.01

*** p < 0.001.

a “other regions” combines data for all states listed in Table 1 except for Uttar Pradesh and Bihar.

## Footnotes

2 For mothers who had more than three live births in the five years before the survey, the NFHS collected place of birth for the last three births. In the states we analyze, 0.3% of births in the last five years are missing information on place of birth.

3 Across the seven regions, an average of about 30% of PSUs are dropped from the high-dimensional fixed effects regression described in Equation 2 because there is only one birth in that PSU. Dropping singleton observations is useful in this context because, if they are not dropped, standard errors will be underestimated (Correia 2016).

4 We include indicator variables that control for every combination of ownership of radio, television, refrigerator, bicycle, motorcycle, telephone, and car, as well as type of flooring (in 15 categories), type of toilet facility (in 13 categories), household electrification, and mother’s educational attainment (in six categories).

5 If we instead run the regressions separately by sex, we get a similar pattern of results. Results are more attenuated for girls, who are biologically more robust than boys. The only surprising result is that in the northwest region, facility birth is statistically significantly associated with survival for girls, but not boys.

6 Another reason why the coefficient may be attenuated in these results is that fewer births are used to identify the association in Equation 2 than Equation 1.

7 See Coffey and Spears (2018) for a discussion of why birth order and sibsize are highly predictive of NNM in India.
